# Effect of the Long COVID-19 Quarantine and Associated Lack of Physical Activity on Overall Health

**DOI:** 10.7759/cureus.30955

**Published:** 2022-11-01

**Authors:** Nahla Hariri, Walaa Takrooni, Nervana Nasraldin, Nizar Bawahab, Enas Alfalogy

**Affiliations:** 1 Community Medicine and Pilgrims Healthcare Department, College of Medicine, Umm Al-Qura University, Makkah, SAU; 2 Preventive Medicine Department, Preventive Medicine Residency Program, Ministry of Health, Jeddah, SAU; 3 Physical Education Department, Faculty of Education, Umm Al-Qura University, Makkah, SAU; 4 General Surgery Department, King Faisal Hospital, Ministry of Health, Makkah, SAU; 5 Family Medicine Department, Faculty of Medicine, Suez Canal University, Ismailia, EGY

**Keywords:** saudi arabia, dietary changes, physical activity, covid-19, quarantine

## Abstract

Background

The COVID-19 pandemic’s mandatory quarantine encouraged a sedentary lifestyle, which had detrimental effects on health. The purpose of this study is to evaluate the health effects of the prolonged COVID-19 quarantine.

Methods

A descriptive cross-sectional study was conducted using an online questionnaire to assess the effects of physical activity and dietary patterns on weight gain and perceived stress.

Results

Of the 384 participants, the majority (58.6%) experienced stress, while 22.4% saw a dramatic decline in physical activity and only 19.8% performed regular physical activity. Although 64.1% of the participants reported eating healthily, 40.6% gained weight during the quarantine. Insufficient exercise (OR 1.966, 95% CI: 1.001-3.858) and consuming soft drinks frequently (OR 2.363, 95% CI: 1.137-4.910) were the two most important predictors of weight gain. The likelihood of developing psychological stress was increased by consuming more food (OR 2.592, 95% CI; 1.268-5.298), eating few vegetables (OR 3.154, 95% CI: 1.203-8.269), and insufficient exercising (OR 2.211, 95% CI: 1.063-4.600).

Conclusion

Long quarantines and physical inactivity raise the risk of weight gain and stress, both of which have detrimental effects on general health.

## Introduction

The first case of the novel coronavirus illness was discovered in China, in December 2019. The Coronavirus Disease 2019 (COVID-19) was declared as an infectious disease induced by the SARS-CoV-2 virus, on February 12, 2020, by World Health Organization (WHO) [1]. A public health concern has emerged because of COVID-19's speedy global dissemination. It had a significant burden in terms of morbidity and mortality as there were over 600 million cases and over six million fatalities worldwide [2]. On March 2, 2020, COVID-19 infection started to appear in Saudi Arabia [3]. To help the public prevent the spread of the disease, preventive measures like wearing masks, washing hands, and avoiding social contact were recommended [2]. A rigorous quarantine was also implemented that required all movement to be restricted to prevent the COVID-19 infection from spreading.

Tackling the spread of the disease by a strict quarantine was a public health priority, particularly at the start of the pandemic when information on the infection's mode of transmission was insufficient and the disease spread quickly among the communities, especially since there were no vaccines available. During the pandemic, few guidelines were provided to the public to raise awareness about maintaining physical activity routines [2]. Long periods of home quarantine as a preventive measure may have led to unhealthy consequences due to a lack of physical activity. The WHO recommends that adults engage in 150 minutes of moderate-to-intense exercise weekly to maintain their health and prevent multiple chronic diseases [4]. For a long time, sedentary behaviors such as lying down and carrying out on-screen activities (e.g., watching TV, using mobiles, and playing video games) may increase the risk of developing diseases [2].

Further, studies [5,6] have shown that physical inactivity during quarantine and an inactive lifestyle led to decreased physical and psychological well-being. Poor well-being has historically been associated with decreased social contact, feelings of isolation, and concern about catching or transmitting illnesses [7,8]. The quarantine periods of previous disease outbreaks have been demonstrated to have detrimental psychological impacts [9]. Psychological symptoms such as stress, worry, and sadness have been linked to periods of lockdown [10]. Healthy behaviors including drinking, eating, sleeping, and exercising were also linked to psychological and mental health during the COVID-19 lockdown [11]. Therefore, this study aims to evaluate the health effects of the prolonged COVID-19 quarantine.

## Materials and methods

Setting

This research was conducted by distributing an online questionnaire created on Google Forms, which was accessible from any device via social media. It was approved by the Medical Ethics Committee at Umm Al-Qura University (no. HAPO-02-K-012-2020-06-395). All the participants were asked to sign the consent form.

Study design and population

This study was a descriptive cross-sectional study. Inclusion criteria included all residents of Makkah city, Saudi Arabia who were above the age of 18 years. The calculated sample size was n = 384 using the common formula for cross-sectional studies that include the population size, z-score, margin of error, and standard deviation: n = [Z2 α/2P (1-P)]/d2, where Z = the desired confidence level (Z = 1.96 for a 95% CI), P = the assumed frequency of the outcome in the population (P = 0.5), and d = precision (d = 0.05) [12].

A self-reported questionnaire was created to gather demographic data and lifestyle characteristics during the quarantine. It entailed four parts. In part one, we asked questions about socio-demographic data such as age, gender, marital status, nationality, history of chronic diseases, smoking, and sleep pattern. In the second part, we asked questions assessing physical activity, including any change in physical activity, type, duration, frequency, available tools, and area for exercising. In the third part, we asked questions about diet, including the number of meals, vegetable intake, fruit intake, soft drink intake, and weight gain. In the fourth part, we asked questions about stress. The Perceived Stress Scale, a well-known tool for identifying how diverse situations affect our emotions and degree of stress, was used to measure stress [13,14]. In this test, the participants responded to 10 questions on their thoughts and feelings over the last four weeks. Each question offered five options for answers: 0 = never, 1 = rarely, 2 = seldom, 3 = frequently, and 4 = usually. The stress score was then calculated. A score between 0 to 13 was considered to be low stress. Moderate stress was defined as a score between 14 and 26. High perceived stress levels were defined as scores between 27 and 40.

Data analysis

IBM SPSS version 25 (IBM Corp., Armonk, NY) was used for the data analysis. For the categorical data, descriptive statistics were used in the form of frequencies and percentages; for the quantitative data, the mean and standard deviation were used if the data were normally distributed; otherwise, the median of the quartiles was used. We used the chi-square test to compare the qualitative variables and determine the statistical significance. Regression analysis was used to identify the determinants of weight gain and perceived stress.

## Results

Of the 384 participants, 56% were women, 59.4% were over 40, 95.6% were Saudis, 4.9% smoked, 18% had chronic illnesses, 70.3% were married, 25.8% were single, and 3.4% were divorced. The majority, 58.6%, experienced stress during the COVID-19 quarantine, 47.7% reported moderated stress levels (their perceived stress scores were between 14 and 26) and 10.9% had high perceived stress levels (their perceived stress scores were between 27 and 40). The sociodemographic data of the participants are described in Table 1.

**Table 1:** Sociodemographic characteristics of the participants

Sociodemographic characteristic	Category	N (%)
Gender	Female	212 (55.2%)
	Male	172 (44.8%)
Age in years	< 20	2 (0.5%)
	20–25	52 (13.5%)
	26–29	62 (16.1%)
	30–39	34 (8.9%)
	≥ 40	228 (59.4%)
Nationality	Saudi	367 (95.6%)
	Non-Saudi	17 (4.4%)
Marital status	Single	99 (25.8%)
	Married	270 (70.3%)
	Divorced	13 (3.4%)
	Widowed	2 (0.5%)
Smoking	Yes	19 (4.9%)
	No	365 (95.1%)
Sleep	Sufficient	225 (58.6%)
	Insufficient	159 (41.4%)
Chronic diseases	Yes	69 (18%)
	No	315 (82%)
Perceived stress	Low level	159 (41.4%)
	Moderate level	183 (47.7%)
	High level	42 (10.9%)

Patterns of physical activity during the quarantine

**Figure 1:**
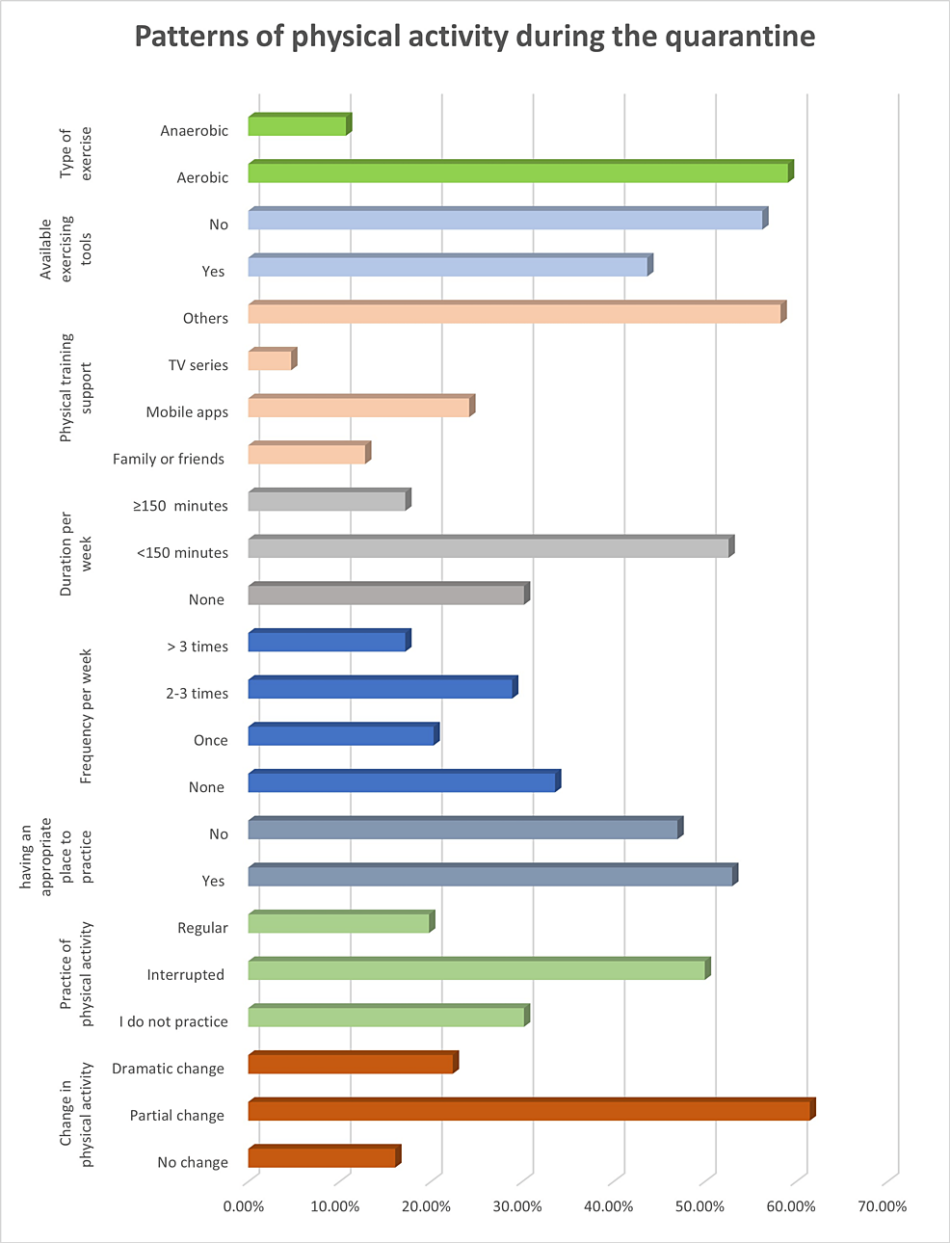
Pattern of physical activity during the quarantine.

The patterns of physical activity during the quarantine are shown in Figure 1. This figure shows that 22.4% of the participants had a dramatic change, 61.5% noted a partial change, and only 16.1% did not notice any changes. The participants displayed a variety of behaviors: 50% engaged in interrupted exercising, while regular physical activity was practiced by 19.8%, and no physical activity was practiced by 30.2% of the participants. Altogether, 43.7% of the respondents possessed the tools to promote exercise. However, while the majority (53%) had a convenient location at home to exercise, only 17.2% did so more than three times and for more than 150 minutes each week. Just 12.8% of the participants went to group exercises with a relative or friend. Mobile training apps were used by 24.2% of the participants, while 59.1% favored aerobic exercise in the different exercise categories.

Patterns of dietary changes during the quarantine

**Figure 2:**
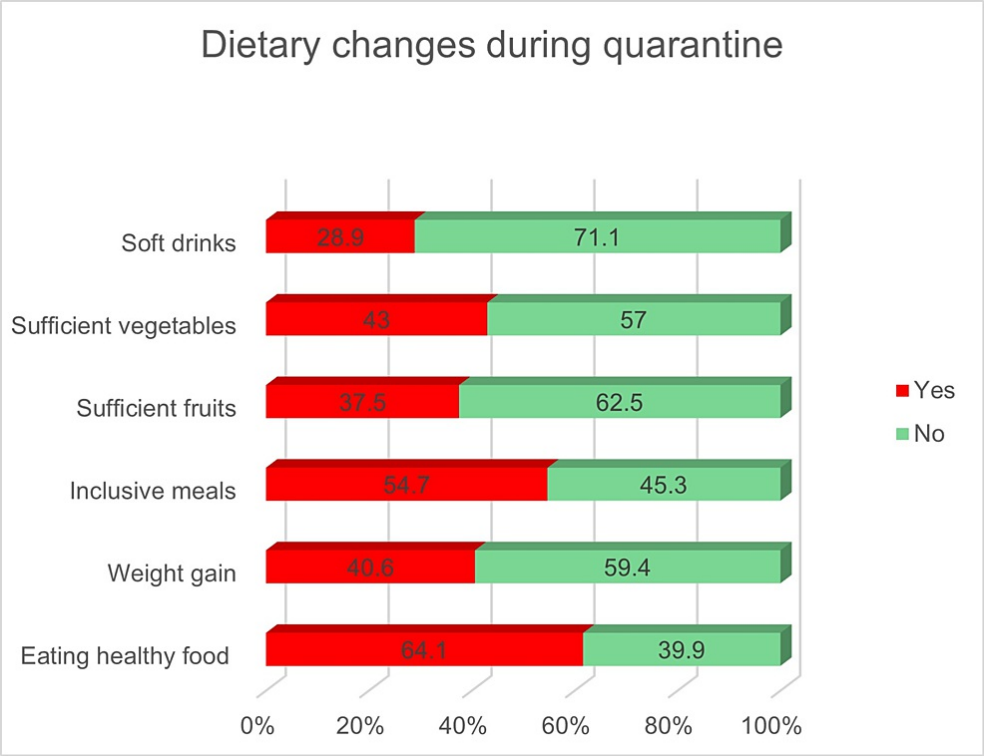
Dietary changes during the quarantine

The patterns of dietary changes during the quarantine are described in Figure 2. Although 64.1% of the participants reported eating healthily, 40.6% gained weight during the quarantine, 54.7% considered their meals to be all-inclusive, 43% reported eating enough vegetables at least three times each week, 37.5% reported eating sufficient fruit more than three times a week, and 28.9% reported consuming soft drinks at least three times each week.

Relation of lifestyle characteristics during a long quarantine with weight gain and stress

Table 2 shows the inferential analysis using the chi-square test to find the association between lifestyle and demographic factors and weight gain and stress. Regarding the risk factors of weight gain, there were significant relationships between increased body weight and age, smoking, change in physical activity, regularity of physical activity, and unhealthy food consumption. Regarding the risk factors of stress, there were significant relationships between increased stress and age, change in physical activity, and regularity of physical activity.

**Table 2:** Relation of lifestyle characteristics of the long quarantine with weight gain and stress

Risk factors of weight gain	Weight gain during the quarantine		Total	X^2^	P value
	Yes	No			
Age in years					
< 20	0 (0.0%)	3 (1.3%)	3 (0.8%)	43.073	0.000
20–25	13 (8.3%)	40 (17.5%)	53 (13.8%)		
26–29	20 (12.8%)	44 (19.3%)	64 (16.7%)		
30–39	2 (1.3%)	34 (14.9%)	36 (9.4%)		
≥ 40	121 (77.6%)	107 (46.9%)	228 (59.4%)		
Change in physical activity					
No change	13 (8.3%)	49 (21.5%)	62 (16.1%)	22.633	0.000
Partial	92 (59.0%)	144 (63.2%)	236 (61.5%)		
Dramatic	51 (32.7%)	35 (15.4%)	86 (22.4%)		
Practice of physical activity					
Regular	18 (11.5%)	58 (25.4%)	76 (19.8%)	11.334	0.003
Interrupted	85 (54.5%)	107 (46.9%)	192 (50.0%)		
No physical activity	53 (34.0%)	63 (27.6%)	116 (30.2%)		
Eating habits					
Healthy	79 (50.6%)	167 (73.2%)	246 (64.1%)	20.557	0.000
Unhealthy	77 (49.4%)	61 (26.8%)	138 (35.9%)		
Smoking	8 (5.8%)	0 (0%)	8 (2.2%)	13.437	0.000
Risk factors of stress	Stress during the quarantine		Total	X^2^	P value
	Yes	No			
Age in years					
< 20	2 (0.9%)	1 (0.6%)	3 (0.8%)	12.379	0.015
20–25	26 (11.6%)	27 (17.0%)	53 (13.8%)		
26–29	46 (20.4%)	18 (11.3%)	64 (16.7%)		
30–39	27 (12.0%)	9 (5.7%)	36 (9.4%)		
≥ 40	124 (55.1%)	104 (65.4%)	228 (59.4%)		
Change in physical activity					
No change	29 (12.9%)	33 (20.8%)	62 (16.1%)	11.677	0.003
Partial	133 (59.1%)	103 (64.8%)	236 (61.5%)		
Dramatic	63 (28.0%)	23 (14.5%)	86 (22.4%)		
Practice of physical activity					
Regular	124 (55.1%)	68 (42.8%)	192 (50.0%)	7.099	0.029
Interrupted	85 (54.5%)	107 (46.9%)	192 (50.0%)		
No physical activity	65 (28.9%)	51 (32.1%)	116 (30.2%)		

Predictors of weight gain and stress

The results of the regression analysis for weight gain and stress are shown in Table 3. This table shows that being a younger age (OR 0.546, 0.337-0.885), eating healthy foods (OR 0.049, 0.011-0.217), and eating fewer meals (OR 0.522, 0.316-0.863) all decreased the risk of weight gain. By contrast, insufficient exercise (OR 1.966, 1.001-3.858) and consuming many soft drinks (OR 2.363, 1.137-4.910) were found to be the two most important predictors of weight gain. Regarding the binary logistic regression analysis for stress, the likelihood of developing psychological stress was increased by consuming more food (OR 2.592, 1.268-5.298), eating few vegetables (OR 3.154, 1.203-8.269), and irregular and insufficient exercising (OR 2.211, 1.063-4.600).

**Table 3:** Binary logistic regression analysis for the predictors of weight gain and stress

Predictor of weight gain	Significance	OR	95% CI lower	95% CI upper
Age	0.014	0.546	0.337	0.885
Duration of practicing physical activity	0.050	1.966	1.001	3.858
Eating healthy food	0.000	0.049	0.011	0.217
No. of daily meals	0.011	0.522	0.316	0.863
Soft drinks	0.021	2.363	1.137	4.910
Predictor of stress	Significance	OR	95% CI Lower	95% CI Upper
Frequency of practicing physical activity	0.034	2.211	1.063	4.600
No. of daily meals	0.009	2.592	1.268	5.298
Low vegetable intake	0.019	3.154	1.203	8.269

## Discussion

The current study evaluated the effects of the prolonged COVID-19 quarantine and associated inactivity on Saudi Arabia’s general health. The findings showed that 30.2% of the respondents did not participate in any physical activity at all during the quarantine, 61.5% experienced a partial reduction in their level of physical activity, and 22.4% had a dramatic shift in their physical activity. This could be because only 53% of the participants had a suitable home exercise area during the quarantine. The findings are consistent with the results of a previous study that found that 52% of the Saudi population in Jeddah drastically decreased their physical activity during the quarantine [15].

A lack of exercise can harm one’s physical and mental health and raise the likelihood of experiencing stress, depression, and anxiety [16]. Promoting physical activity may have been challenging during the COVID-19 pandemic [17]. According to the results of the present study, only 17.2% of the participants engaged in adequate physical exercise lasting more than 150 minutes per week. Despite the availability of various mobile apps, only 24.2% of the participants used them to perform physical training sessions and 12.8% carried out physical training sessions with family and friends. Another study found that group training helps people practice efficient and continued exercise and highlights the value of physical literacy, the importance of exercise, and improving adapting mechanisms, particularly under stressful conditions [18].

Regarding dietary changes during the quarantine, 64% of the participants reported eating healthily; however, 40.6% gained weight, which is greater than the estimate provided by previous research carried out in the United Arab Emirates, which found that 31% of their subjects gained weight [19]. Another study showed that 28% gained weight in Jeddah owing to being sedentary during the quarantine [15]. This might be due to the multifactorial nature of weight gain, which is not only related to dietary factors, but also a lack of activities, social isolation, mood changes, and inappropriate sleep patterns, as only 17% of the participants had adequate sleep. Weight gain is significantly related to age, smoking, irregular physical activity, and eating unhealthy food. A similar significant relation was detected by another study conducted in the US, as weight gain was related to an unhealthy diet, frequent snacking, irregular physical activity, and stress [20]. Our logistic regression analysis showed that being a younger age, eating healthy foods, and eating fewer meals were inversely associated with weight gain. By contrast, performing insufficient exercise and consuming many soft drinks were found to be the two most important significant risk factors of weight gain. The results are in line with a Chinese study that identified excessive food consumption and inadequate exercise as the two main risk factors of weight gain [21].

The prolonged COVID-19 quarantine had a significant impact on mental health as well as general health. The current study found that 47.7% of the participants reported moderate levels of perceived stress and 10.9% reported high perceived stress levels. These findings are similar to the results revealed in a Filipino study [22], which showed that 28.8% had symptoms suggestive of anxiety, 16.9% had depressive symptoms, and 13.4% developed psychological stress. According to the results of our multivariate logistic regression analysis, psychological stress was predicted by consuming more food, eating few vegetables, and irregular and insufficient exercising.

## Conclusions

The long quarantine and low physical inactivity during the COVID-19 pandemic increased the risk of weight gain and perceived stress, both of which lower general health markedly. It is advised that a national strategy be formulated to promote physical activity and discourage a sedentary lifestyle. The promotion of physical activity awareness and the encouragement and adoption of healthy lifestyle habits among all Saudi citizens are significant goals for healthcare providers.
